# Conversion mechanism of heptachlor by a novel bacterial strain

**DOI:** 10.1039/c7ra10097c

**Published:** 2018-02-02

**Authors:** Liping Qiu, Hu Wang, Xuntao Wang

**Affiliations:** Key Laboratory of Subsurface Hydrology and Ecological Effects in Arid Regio, Ministry of Education, School of Environmental Science and Engineering, Chang'an University Xi'an 710054 P. R. China lpqiu3699@sina.com +86-29-80334563 +81-29-80334563; School of Natural and Applied Sciences, Chang'an University Xi'an 710064 P. R. China

## Abstract

Microbial treatment is the preferred method for the remediation of soil and water contaminated by heptachlor. We collected sludge samples from the sewage biological treatment pool of Shaanxi Insecticide Factory in Xi'an, China, which were used as bacteria source. With heptachlor as the substrate, at 30–35 °C, an effective microorganism (named strain H) for heptachlor degradation was isolated successfully after a long period of acclimation, screening and purification. Strain H was able to use heptachlor as a carbon source and had a good capacity for biodegradation of heptachlor. Strain H was preliminarily identified as a Gram-negative, short rod-shaped, single-cell bacterial strain that was similar to the genus Escherichia or Shigella, according to the analysis of its morphology and physiological–biochemical characteristics. Then, strain H was further identified as a novel bacterium based on the similarity analysis of its 16S rDNA gene sequence with the sequences logged in the RDP and GenBank databases. The 16S rDNA of this bacterium has never been reported before. When the inoculation volume and the pH were 20% and 7.1–7.6, respectively, the degradation rate of heptachlor can reach more than 88.2% in 130 h, with the initial concentration of heptachlor being 300 μg L^−1^ at 30–35 °C. Identification of the metabolites by GC/MS showed that strain H degrades heptachlor *via* two pathways simultaneously, *i.e.*, pathway (1) hydroxylation at the C1 position of heptachlor to 1-hydroxychlordene followed by epoxidation and dechlorination to chlordene epoxide; and pathway (2) epoxidation at the C2 and C3 positions of heptachlor to heptachlor epoxide, and then heptachlor epoxide was further transformed to chlordene epoxide by dechlorination reaction, or degraded to heptachlor diol by hydrolysis reaction. The biodegradation of heptachlor indicated that heptachlor and its metabolites can be converted into less-toxic small molecular metabolites by a series of reactions such as epoxidation, hydrolysis and dechlorination reactions.

## Abstract 2

Microbial treatment is the preferred method for the remediation of soil and water contaminated by heptachlor.

## Graphical abstract

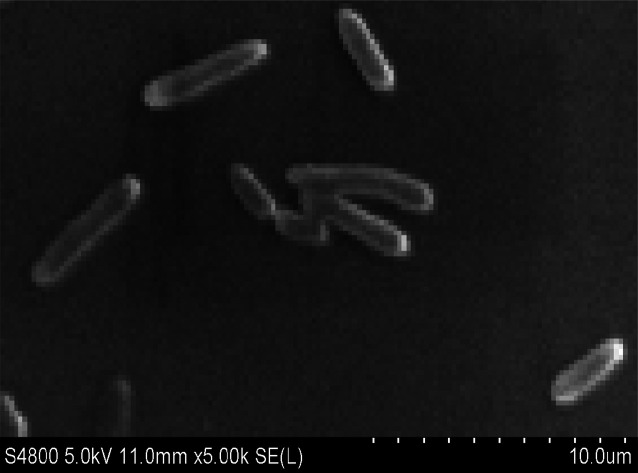

## Introduction

Heptachlor (C_10_H_5_Cl_7_) is an organochlorine cyclodiene insecticide, first isolated from technical chlordane in 1946. During the 1960s and 1970s, it was used primarily by farmers to kill termites, ants, and soil insects in seed grains and on crops, as well as by exterminators and home owners to kill termites. Heptachlor is a highly to moderately toxic compound in EPA toxicity class II. In 1988, the EPA canceled all uses of heptachlor in the U.S. The only commercial use still permitted is for fire ant control in power transformers.^1^ Heptachlor is still available outside the U.S.^2^ For example, some developing countries continue to use this pesticide in both agriculture and public health programs because of its low cost and versatility in controlling various pests.^3^ Before heptachlor was banned, formulations available included dusts, wettable powders, emulsifiable concentrates, and oil solutions. It acts as a nonsystemic stomach and contact insecticide.^4^

The use of microbial degradation is one of the most effective ways to remove pollutants in the environment because it enjoys less chemical agents, lower energy consumption, lower cost, milder reaction condition and less secondary pollution compared to physicochemical methods for heptachlor treatment. Microbiological treatment technology of organochlorine pesticides pollution has become an important research direction. At present, the study of heptachlor concentrates on the distribution characteristics and toxic effects in the environment, while few studies have been conducted on microbial degradation.^5–7^ We isolated a novel bacterial strain that can grow using heptachlor as the carbon source and has high degradation efficiency from the sludge of sewage biological treatment pool in chemical plant. Subsequently, we carried out the identification of physiological and biochemical characteristics for the isolate, and the identification of its 16S rDNA gene sequence. The metabolites of heptachlor were analyzed through GC/MS, and search matching with the NIST spectral library, as well as comparison with standard compounds. On this basis, we studied the bioconversion mechanisms and degradation pathways of heptachlor. This study is expected to provide a good microbiological source, as well as theoretical basis for biodegradation of soil and water contaminated by heptachlor in the future.

## Materials and methods

### Materials

The mineral salt medium (MSM) contained (g L^−1^): 0.5 g NH_4_NO_3_, 1.5 K_2_HPO_4_·3H_2_O, 1.0 MgSO_4_·7H_2_O, 0.03 FeSO_4_·7H_2_O, 1.0 NaCl and 2 mL of trace elements solution which contained (g L^−1^): 0.2 ZnSO_4_·7H_2_O, 2.0 CaCl_2_·2H_2_O, 0.2 MnSO_4_·4H_2_O, 0.1 CuSO_4_·2H_2_O, 0.12 CoCl_2_·6H_2_O, 0.12 Na_2_MoO_4_·2H_2_O and 0.10 H_3_BO_3_. Luria–Bertani (LB) liquid medium contained the following ingredients (g L^−1^): 10.0 peptone, 5.0 sodium chloride, 10.0 yeast extract.

Heptachlor standard (1 mg mL^−1^ in methanol) and heptachlor epoxide standard (1 mg mL^−1^ in methanol) were obtained from Aladdin Industrial Corporation (Shanghai, China). 1-Hydroxychlordene standard (10 μg mL^−1^ in cyclohexane) was obtained from Dr Ehrenstorfer GmbH, Germany. *N*-Hexane (chromatographically pure solvents) was obtained from American TEDIA Company.

All other chemical reagents used in the experiments were of analytical reagent grade.

### Isolation of the heptachlor-degrading bacterium

Sludge samples were taken from the sludge of sewage biological treatment pool of Shaanxi Insecticide Factory in Xi'an, China in April 2016. The basic operating parameters of sewage biological treatment are as follows: 200 m^3^ h^−1^ of influent water, 1.23 kg [CODcr] per (m^3^ d) of volume load, 25–30 °C of operating temperature, A^2^/O systems. Sludge volume index (SVI) 130–150 mL g^−1^. The initial enrichment culture was established in a 250 mL Erlenmeyer flask by inoculating 200 mL sterile MSM containing 100 μg L^−1^ heptachlor with 10 g sludge samples (wet weight). The Erlenmeyer flask was incubated on a constant-temperature rotary shaking incubator (BS-1E, China) at 30 °C and 120 rpm. After one-week's incubation, portions were inoculated into a fresh sterile MSM with heptachlor concentration up to 300 μg L^−1^, and incubation was continued. After five more transfers at 1 week intervals, the culture was purified by the plate separation method onto solidified MSM containing 300 μg L^−1^ heptachlor. Finally, a pure strain was isolated and named as strain H.

### Identification of strain H

A MOTIC digital biological microscope (DMBA400-P, China) and a scanning electron microscope (S-4800, Hitachi) were used for the observation of the morphology and the size of the bacterium. Physiological and biochemical characteristic of strain H were determined by the procedures described from Bergey's Manual of Determinative Bacteriology^8^ and Manual of Common Bacterial System Identification.^9^

The DNA of the strain H culture was obtained using a commercial genomic DNA extraction kit (SK1201-UNIQ-10, Shanghai Sangon Biotech Co., Ltd., China). 16S rRNA gene of the strain was amplified from the bacterial genomic DNA by a PCR using universal primers of 7F (5′-CAGAGTTTGATCCTGGCT-3′) and R1540 (5′-AGGAGGTGATCCAGCCGCA-3′).^10^ PCR amplification condition was as follows: each PCR mixture (25 μL) was composed of 0.5 μL genomic DNA ((20–50 ng μL^−1^)); 1.0 μL of dNTP at 2.5 mM; 2.5 μL 5× buffer (with 20 mmol L^−1^ Mg^2+^); 0.5 μL forward primer (10 μmol L^−1^), 0.5 μL reverse primer (10 μmol L^−1^), 0.2 μL Taq (5 U μL^−1^), and sterile water. The PCR was performed in a PTC-100 Peltier Thermal Cycler (MJ Research, USA) with a hot starting performed at 98 °C for 5 min, followed by 28 cycles of 98 °C for 30 s, annealing at 55 °C for 45 s, and 72 °C for 1 min, followed by a final extension at 72 °C for 10 min.

The 16S rRNA gene of about 1.5 kb was purified using a gel extraction kit (SK1191-UNIQ-10, Shanghai Sangon Biotech Co., Ltd., China), and the purified product was ligated with pMD 18-T vector. Then the ligation product was transformed into competent bacterial strain JM109, and the positive clones were picked out according to blue/white screening. The recombinant plasmid was extracted from the positive clone according to alkaline lysis method.^11^

The PCR products were purified and sequenced at Shanghai Sangon Biotech Co., Ltd., (China) and compared with 16S rDNA sequence data from type strains available in Genbank (http://www.ncbi.nlm.nih.gov) and the Ribosomal Database Project [RDP] (http://rdp.cme.msu.edu/index.jsp) using the BLASTN and RDP sequence match routines. The sequences were aligned using multiple sequence alignment software CLUSTAL W version 1.81. A phylogenetic tree was then constructed by the neighbor-joining method using the MEGA software (version 4.1) based on the 16S rDNA sequences.

### Growth curve of strain H

We incubated the isolated strain H in a sterilized LB (initial concentration of the strain: 1.2 × 10^8^ cells per mL) in a shaking incubator at 30 ± 0.5 °C and 120 rpm to obtain the growth curve of strain H at different phases by measuring the concentration of strain H periodically.

### Optimal conditions of heptachlor degradation by strain H

Strain H at the logarithmic growth phase was collected by centrifugation. The strain was then resuspended using physiological saline water. The resulting suspension was used as degradation inoculation solution for all the following experiments. Effects of environmental conditions for the growth of strain H and heptachlor degradation were investigated. In each of our experiments, a 250 mL Erlenmeyer flask was used which contains 200 mL sterilized MSM containing 300 μg L^−1^ heptachlor and 20% of inoculation volume with a biomass content of 1.2 × 10^8^ cells per mL, and the degradation time was 140 h. Tests on the effect of temperature were first examined at five levels, 20, 25, 30, 35 and 40 °C. The temperature is automatically set and adjusted by the constant-temperature rotary shaking incubator (BS-1E, China), at pH 7. These were followed by tests on the effect of pH at six levels from pH 5.2 to 9.3. The pH level is adjusted with 10% NaOH solution and 10% HCl solution. The pH level is measured by the pH meter (FE20K, METTLER TOLEDO, Switzerland) at the identified optimal temperature. Experiments on heptachlor degradation were carried at the optimal pH and temperature in 250 mL Erlenmeyer flasks at 120 rpm. Each flask contained 20% of inoculation volume (1.2 × 10^8^ cells per mL) and 200 mL MSM with heptachlor initial concentration varying from 100 to 500 μg L^−1^.

### Metabolites and conversion mechanism of heptachlor

Heptachlor degradation and strain H growth were examined at the identified optimal conditions. The metabolites of heptachlor were qualitatively and quantitatively analyzed by gas chromatography-mass spectrometry (GC/MS2010, Shimadzu Corp.), and comparison with standard compounds and document data. Subsequently, the degradation mechanism of heptachlor by strain H was investigated.

### Concentration determination of heptachlor, its metabolites and strain cells

The concentrations of heptachlor and its metabolites were analyzed by a Shimadzu GC/MS2010 apparatus with a RTX-5MS from Shimadzu (30 m × 0.25 mm × 0.25 mm). The column temperature program was 80 °C isothermal for 5 min and then from 80 °C to 260 °C with an increment of 10 °C min^−1^. The MS energy was derived from a 70 eV electron ionization source of electron bombardment; ion source temperature was maintained at 200 °C; interface temperature was maintained at 260 °C; mass scan range was set at 40–450 *m*/*z*.

Samples were prepared in a 40 mL brown reagent bottle. A volume of 10 mL sample contained heptachlor and its metabolites obtained after the pretreatment of the bacterial cultures was placed into the reagent bottle, and then five milliliter *n*-hexane was added.^12,13^ The bottle was kept in a constant-temperature rotating shaker (HZQ-C, China) at 200 rpm for 20 min and then in ultrasonic waves at 28 kHz frequency for 10 min for heptachlor extraction. The extraction process was repeated three times. The organic fraction was dried over anhydrous sodium sulfate. Finally, 1 μL of the dry organic sample that passed through an organic filter (hydrophobic PTFE) of 0.22 μm pore size was analyzed by GC/MS.

The cell concentrations of all samples were detected using Visible Spectrophotometry and Petroff-Hausser counting chamber.^14^

### Statistical analysis

Excel 2007 and SPSS statistical software were used for data processing and statistical analysis of the experimental results. All the experiments were conducted in triplicate. The relative standard deviation of all the data points in this paper was between 0.72% and 4.63%.

## Results and discussion

### Identification of strain H

**Fig. 1:**
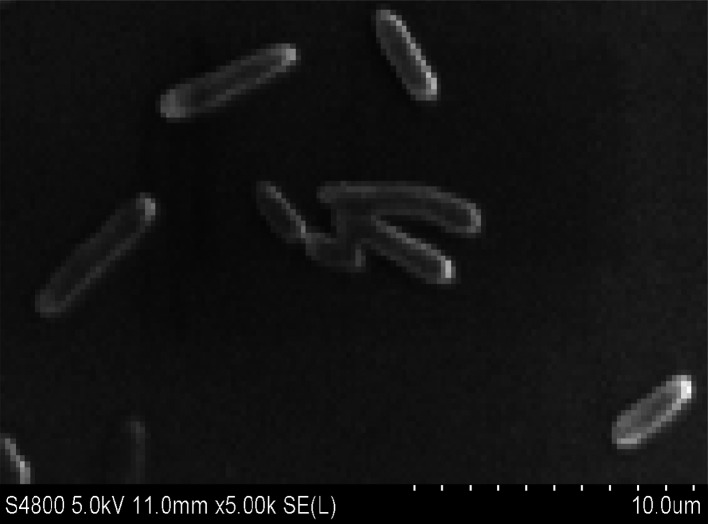
Electronic microscopy photograph of strain H.

Strain H was inoculated to a sterilized solidified MSM also containing 300 μg L^−1^ heptachlor and incubated for 120 h. Through the MOTIC digital biological microscope and a scanning electron microscope (S-4800, Hitachi), strain H was observed as a short rod-shaped, single-cell Gram-negative bacterium (Fig. 1). The colony morphology of strain H showed pale yellow and circular. The diameter of the colony was less than 0.3 cm, smooth on the surface and trim on the edge, and translucent. There was a slight bump in the middle. Methyl red reaction, starch hydrolysis and catalase reaction were positive. V–P reaction, oxidase and gelatin reaction were negative. Phenotypic and physiochemical experiments suggested that strain H was similar to genus Escherichia or genus Shigella.

**Fig. 2:**
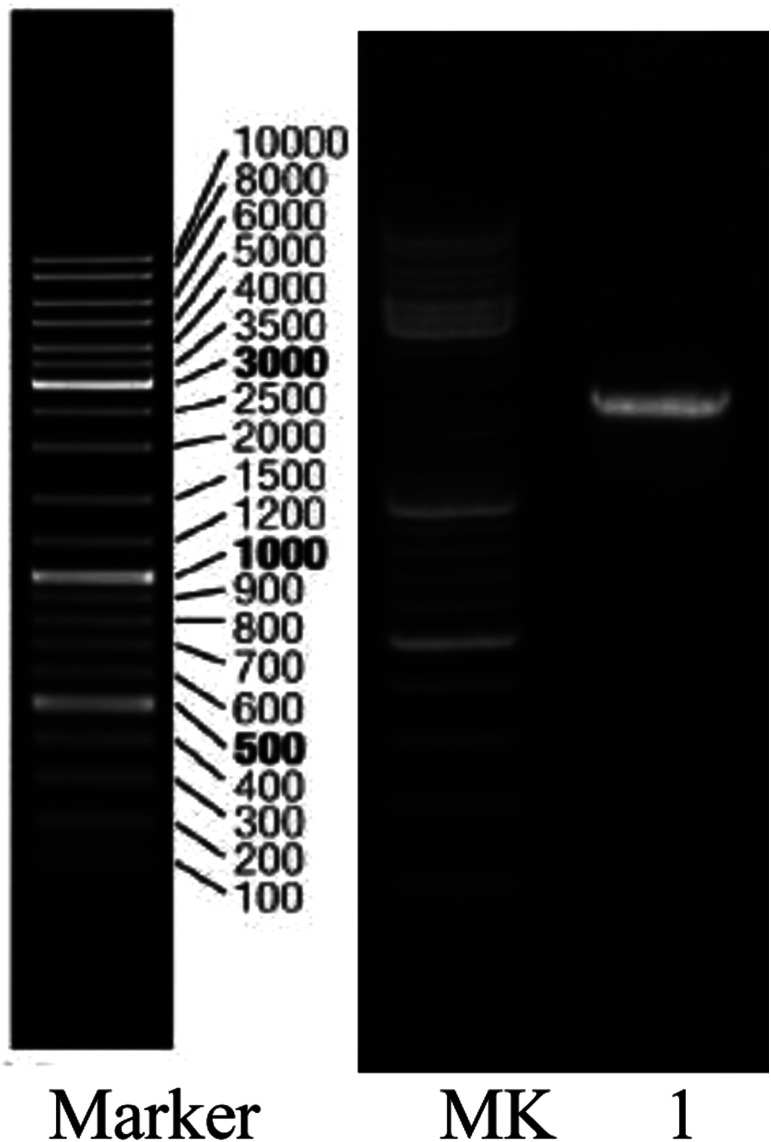
Gel electrophoresis pattern of the PCR amplification product of strain H: lane 1, strain QL; MK, Marker (DL2000).

**Fig. 3:**
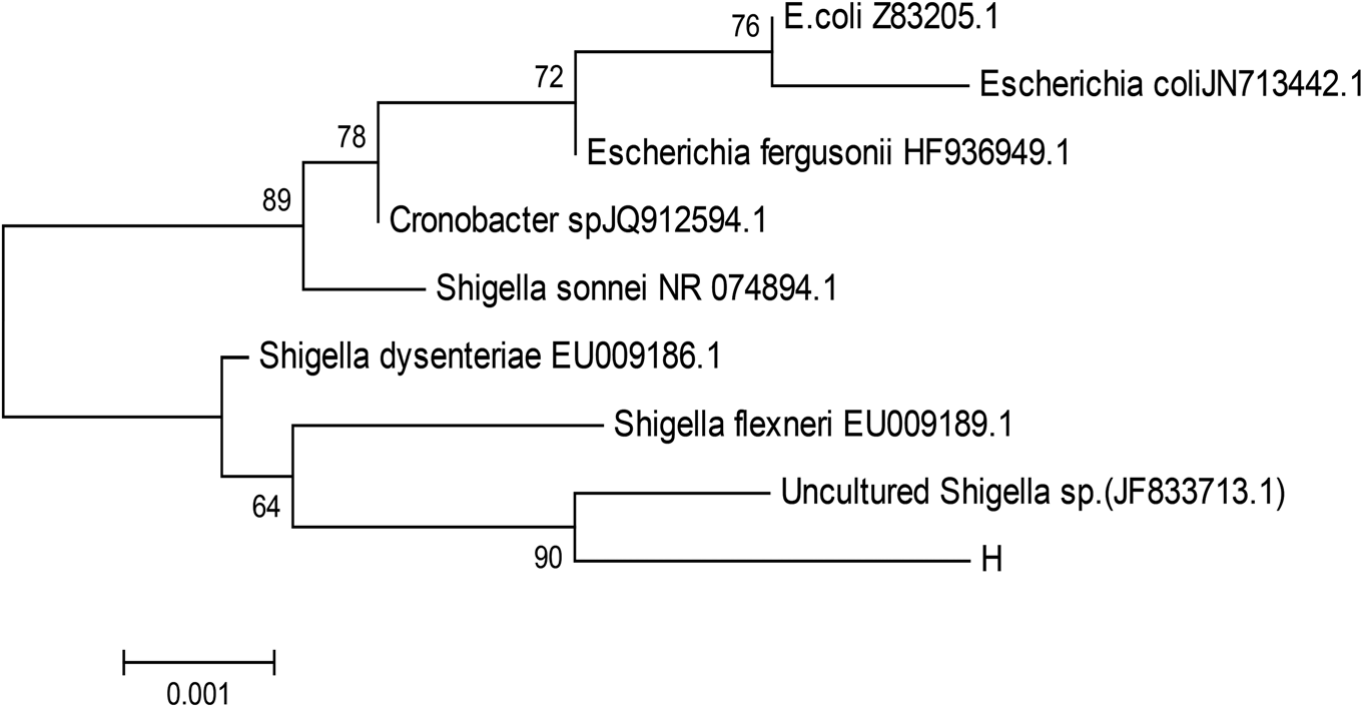
Phylogenetic tree based on 16S rDNA sequences. Numbers at the nodes indicated the percentages of bootstrap samplings, derived from 1000 samples that support the internal branches.

The bacterium was further identified by 16S rDNA analysis. After gel electrophoresis of the PCR amplification product of strain H as shown in Fig. 2, a single band of 1.5 kilobases (kb) could be seen by comparing with the DNA Ladder Mix Marker (DL2000) in the gel imager. The accurate length of the band was determined as 1534 base pairs (bp) after sequencing. The 16S rDNA gene sequence of strain H (accession number: BankIt2073018 Seq1 MG711918) was compared with the sequences logged in the RDP and GenBank databases. Strain H was further identified as a novel bacterium because its homology with all known strains was very small based on homology analysis. The phylogenetic tree of strain H was constructed based on 16S rDNA sequences (Fig. 3).

### Growth curve of the activity strain

**Fig. 4:**
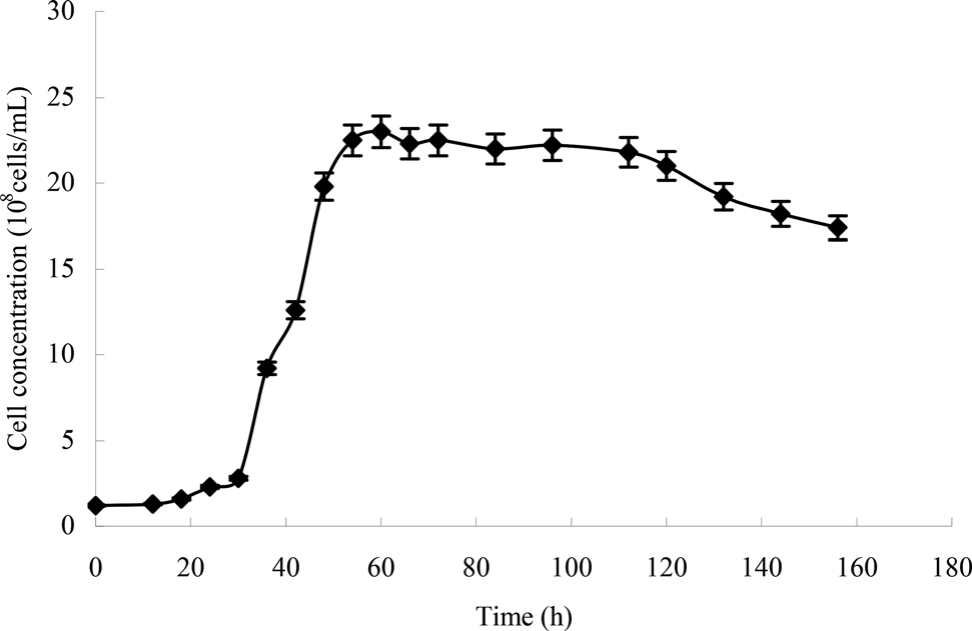
Growth curve of the strain (initial concentration: 1.2 × 10^8^ cells per mL) in LB.

From the growth curve of strain H (Fig. 4), we can see that the lag phase lasted from 0 to 30 h, the logarithmic phase lasted from 30 to 60 h, the stationary phase lasted from 60 to 120 h, and the death phase began at 120 h.

### Optimal conditions of heptachlor degradation by strain H

**Fig. 5:**
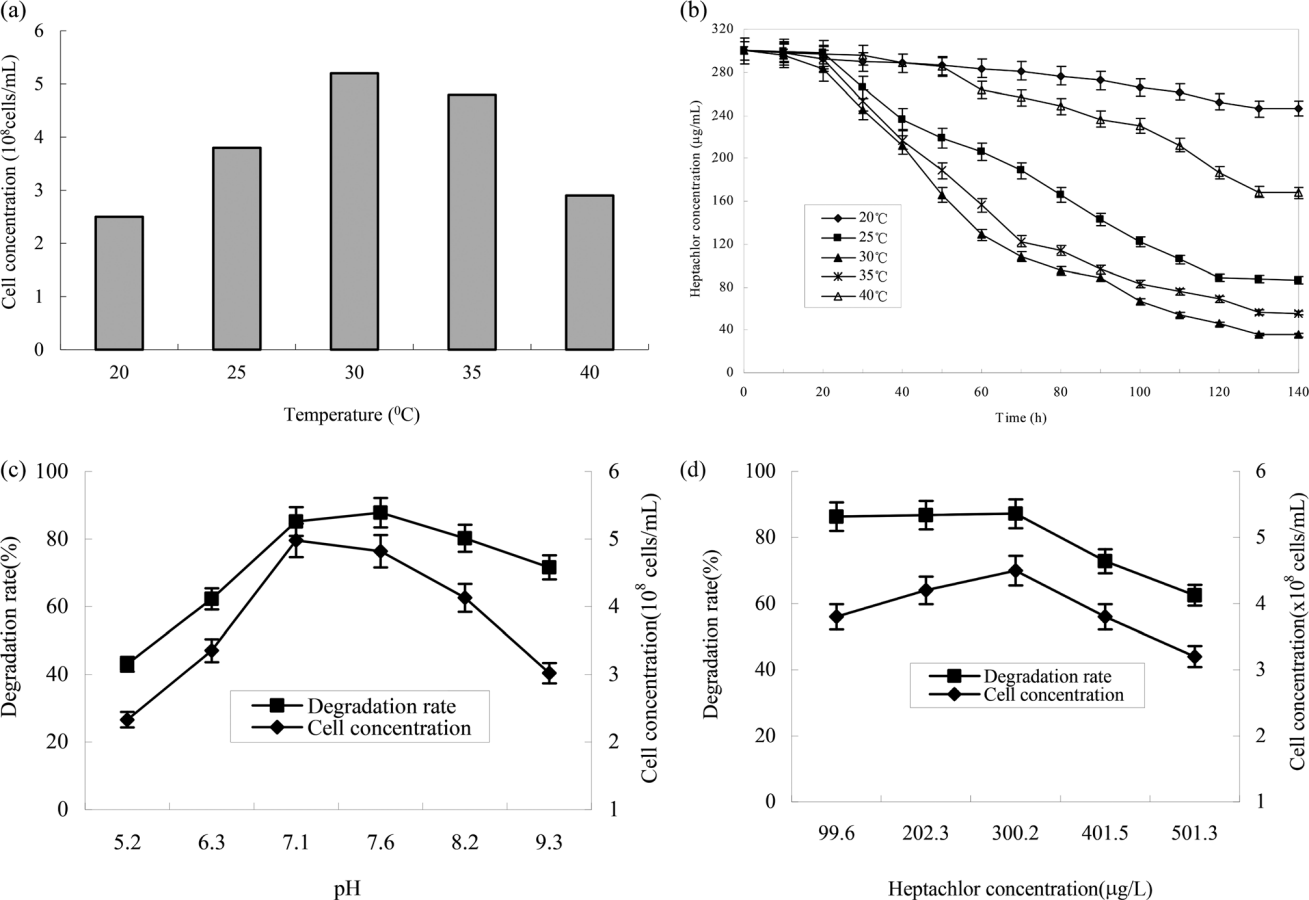
Effects of different conditions on heptachlor degradation characterization by strain H. (a) Effect of temperature on the growth of strain H; (b) effect of temperature on heptachlor degradation by strain H; (c) degradation pH values; (d) initial heptachlor concentrations.

The effect of temperature on the growth of strain H is shown in Fig. 5a and b. The growth of strains would be affected under too high or too low temperature. 30 °C was suitable for the growth of strain H. The degradation rate of heptachlor was poor under the condition of 20 °C and 40 °C. The degradation of heptachlor was better when the temperature was between 25 °C and 35 °C. The degradation rate of heptachlor was the best at 30 °C. The growth curves at different temperature are shown in Fig. 13.

The effect of initial pH value on the degradation of heptachlor is shown in Fig. 5c and 11. The cell concentration of strain H increased quickly when pH increased from 5.2 to 7.1. The highest cell concentration was observed at pH 7.1–7.6. When pH was above 7.6, the cell concentration decreased gently. Under the acidic condition (pH < 6), the degradation rate of heptachlor by strain H was very low. Under the neutral and alkaline conditions, the degradation became high. When pH was 7.6, the degradation rate reached the maximum. We reason that the degrading activity of the enzyme^15^ produced by strain H was low under the acidic conditions, and the degrading activity was higher under neutral and alkaline conditions. When pH was 7.1–7.6, the degrading activity was the highest, so the degradation effect was the best. The growth curves at different pH levels are shown in Fig. 14.

Different heptachlor initial concentrations were assessed as shown in Fig. 5d and 12. It can be seen that strain H had strong degradation capacity of heptachlor, especially at low heptachlor concentration (99.6–202.3 μg L^−1^). When the heptachlor initial concentrations were 99.6 and 202.3 μg L^−1^, strain H could degrade heptachlor 86.3% and 86.8%, respectively. No appreciable loss of heptachlor was observed in the sterile control. 99.6–202.3 μg L^−1^ were heptachlor concentrations detected in most contaminated soil and discharged wastewater,^16^ so the strain has a great advantage of solving practical heptachlor pollution.

The degradation rate of heptachlor increased with increasing heptachlor concentration up to 300.2 μg L^−1^, the largest degradation rate attained 87.2%. When the heptachlor initial concentration was up to 401.5 and 501.3 μg L^−1^, the degradation rate were 75.6% and 62.5%, respectively. This result could be due to the toxicity of heptachlor to the microorganism. Once the heptachlor initial concentration exceeded a certain value, it would inhibit the growth of strain H, thus inhibiting its degradation ability.^17^ The growth curves at different heptachlor concentrations are shown in Fig. 15.

### Metabolites and conversion mechanism of heptachlor

**Fig. 6:**
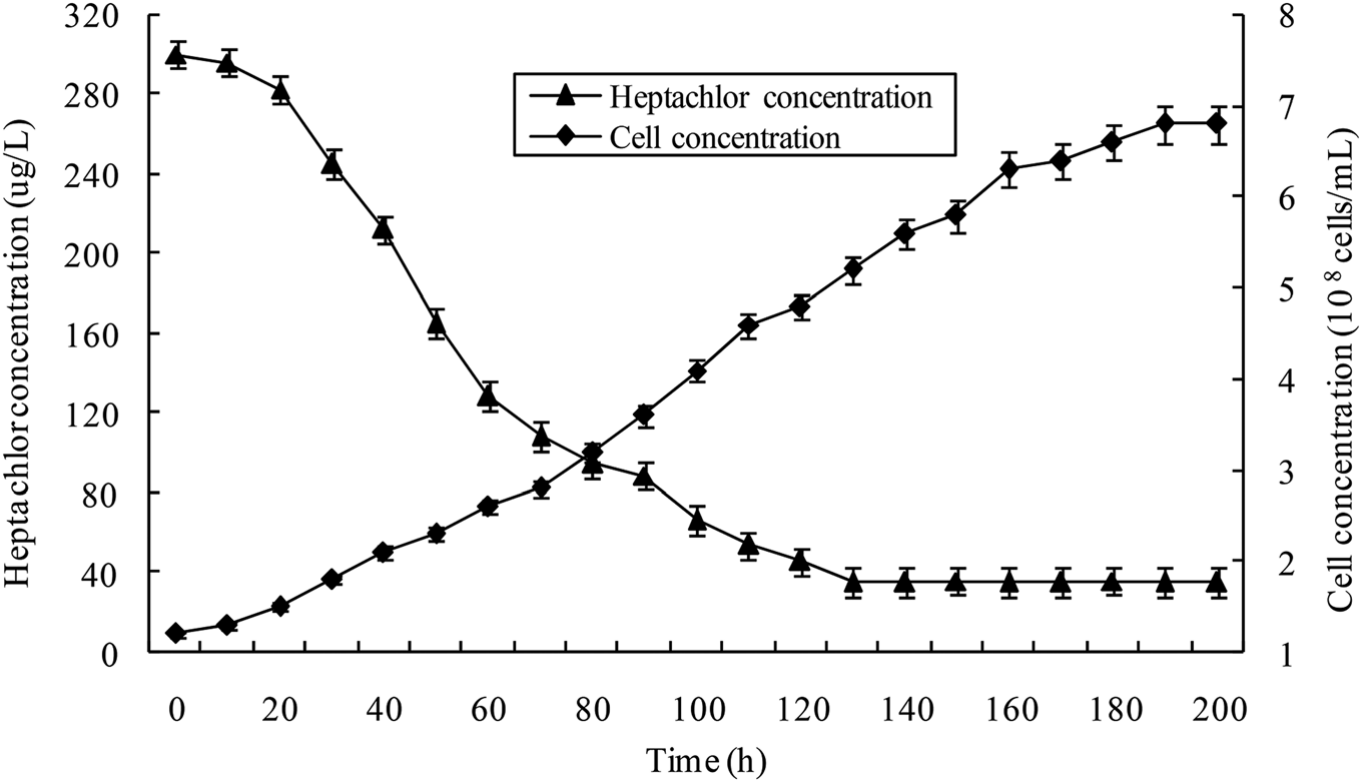
Heptachlor degradation and the growth of strain H at the optimal conditions.

**Fig. 7:**
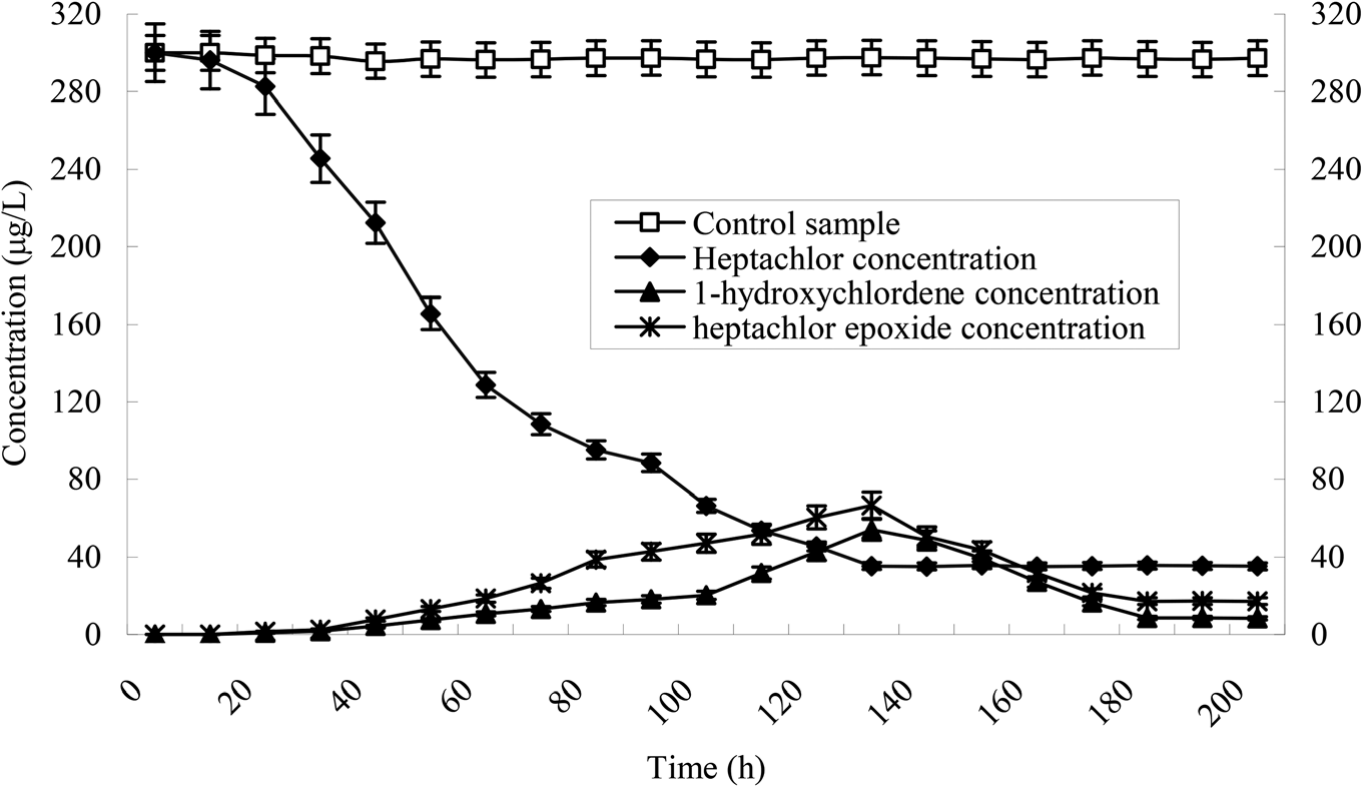
Time course of degradation of heptachlor and production of some metabolites.

Heptachlor biodegradation and the growth of strain H is shown in Fig. 6 under the above identified optimal conditions. The cell concentration of strain H increased as the concentration of heptachlor decreased at the optimal conditions. Even after the heptachlor degradation ability reached the maximum at 130 h, the bacteria continued to grow, which indicates that strain H can grow on some of the degradation products of heptachlor such as 1-hydroxychlordene or heptachlor epoxide. Heptachlor epoxide has similar toxicity with heptachlor, and some studies have shown that certain microorganisms, such as *Phlebia*, can open the oxygen ring structure of heptachlor epoxide by hydrolysis reaction, and that heptachlor epoxide can be further converted into hydroxylated metabolites such as the relatively less-toxic 1-hydroxychlordene. 1-Hydroxychlordene could be metabolized easily into hydrophilic products of even less toxicity.^18–20^ From Fig. 7, we observe that the concentrations of the heptachlor metabolites gradually decreased over time: 1-hydroxychlordene from 54.2 μg L^−1^ to 8.3 μg L^−1^, and heptachlor epoxide from 66.6 μg L^−1^ to 17.2 μg L^−1^, which verified the theory.

**Fig. 8:**
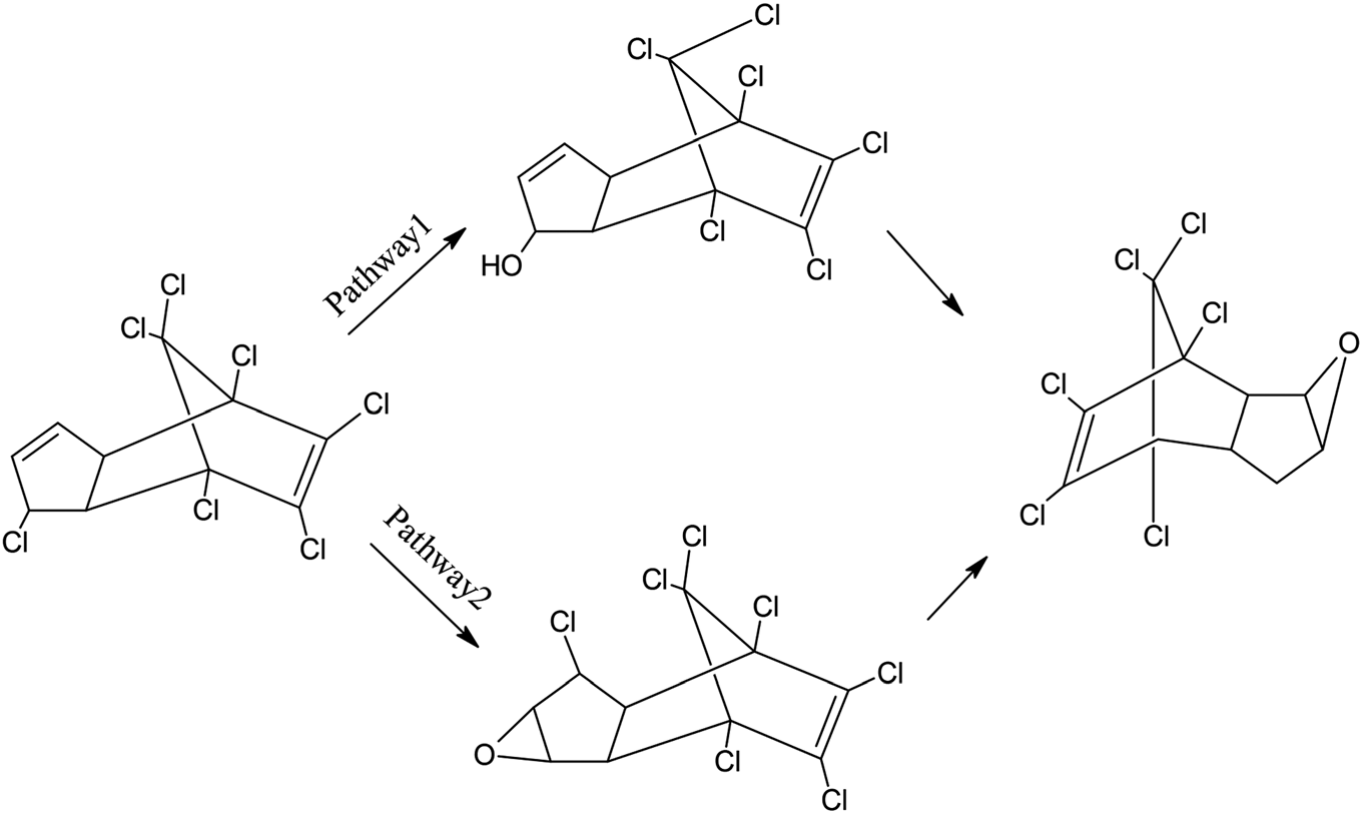
The proposed metabolic pathways of heptachlor by strain H.

**Fig. 9:**
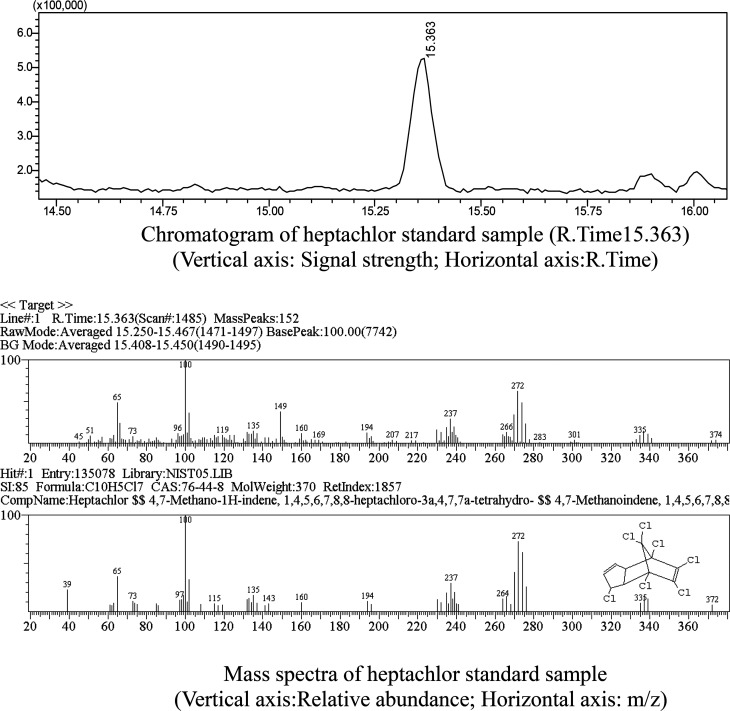
Chromatogram and mass spectra of heptachlor standard sample.

**Fig. 10:**
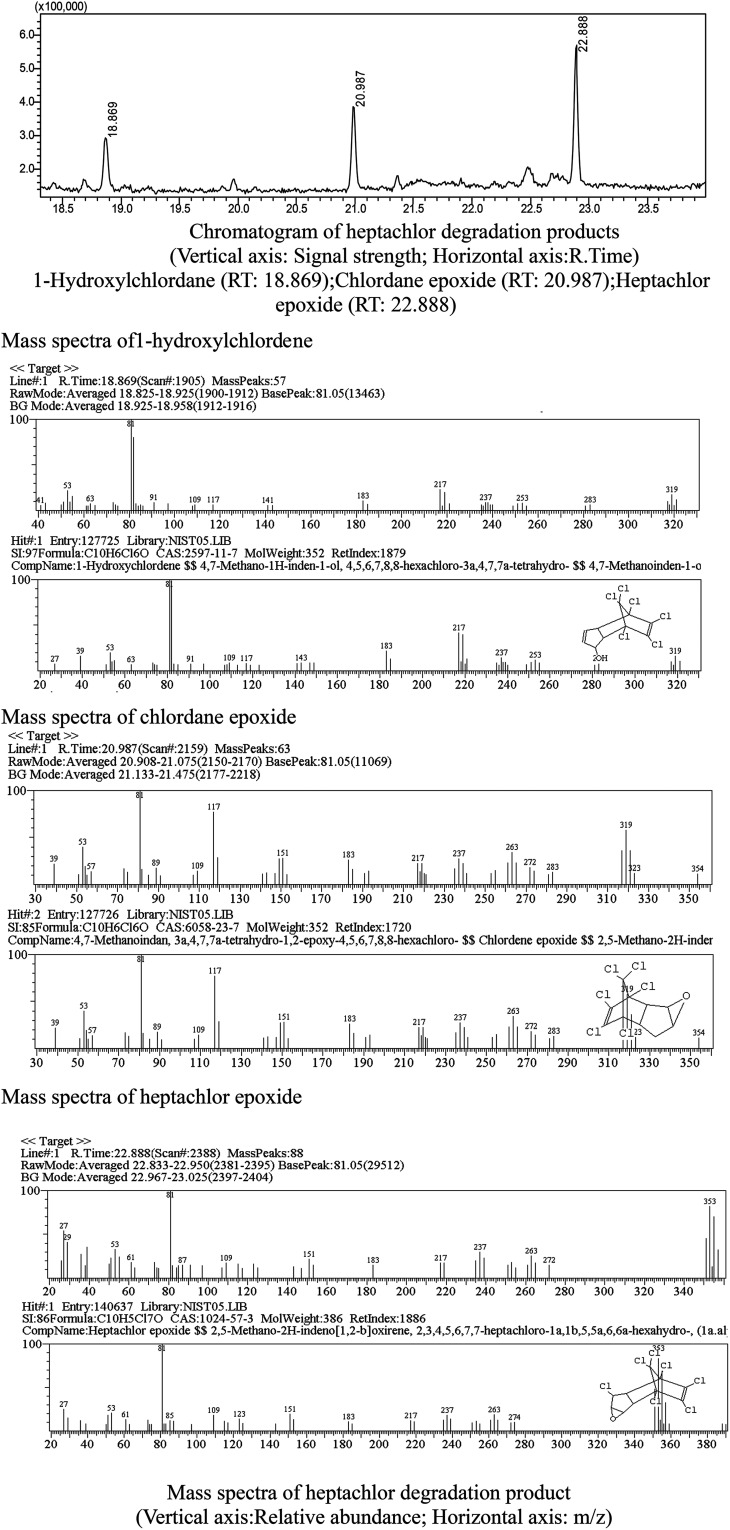
Chromatogram and mass spectra of heptachlor degradation products.

Metabolites and conversion mechanism of heptachlor is shown Fig. 7. During the degradation process, the degradation of heptachlor was accompanied by the production of metabolites. When the heptachlor degradation rate reached the maximum, 88.2% at 130 h, 3 main metabolites were detected in the extracts from the culture medium. Through GC-MS analysis, search matching with the NIST spectral library, and comparison with standard compounds and document data,^2^ the metabolites were identified respectively as 1-hydroxychlordene (R. time 18.869 min), chlordene epoxide (R. time 20.987 min) and heptachlor epoxide (R. time 22.888 min), as shown Fig. 9 and 10. Compared to other trace metabolites, the concentration of 1-hydroxychlordene and heptachlor epoxy detected was relatively high, *i.e.* 54.2 μg L^−1^ and 66.6 μg L^−1^, which was equivalent to 18.1% and 22.2% of the total added concentration of heptachlor respectively.

**Fig. 11:**
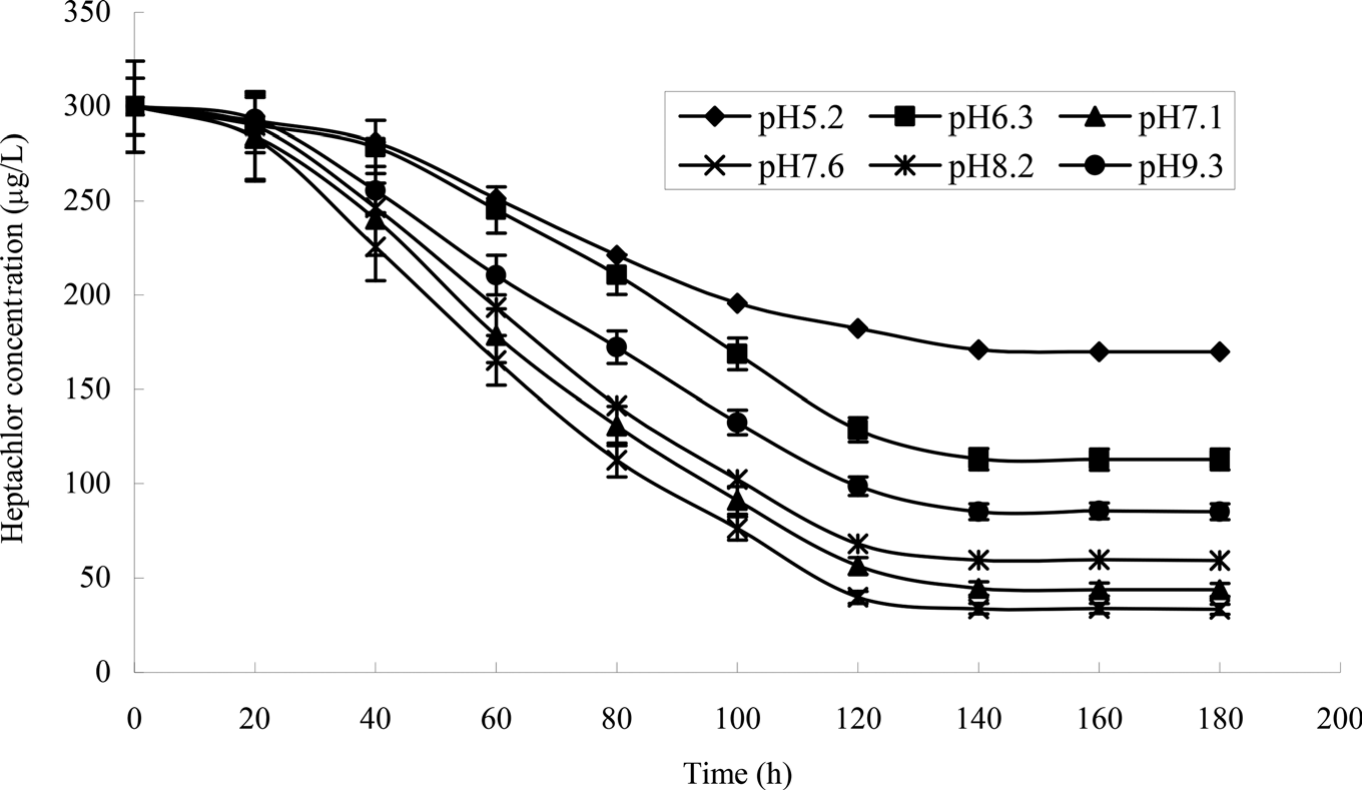
Degradation curves at different pH levels.

**Fig. 12:**
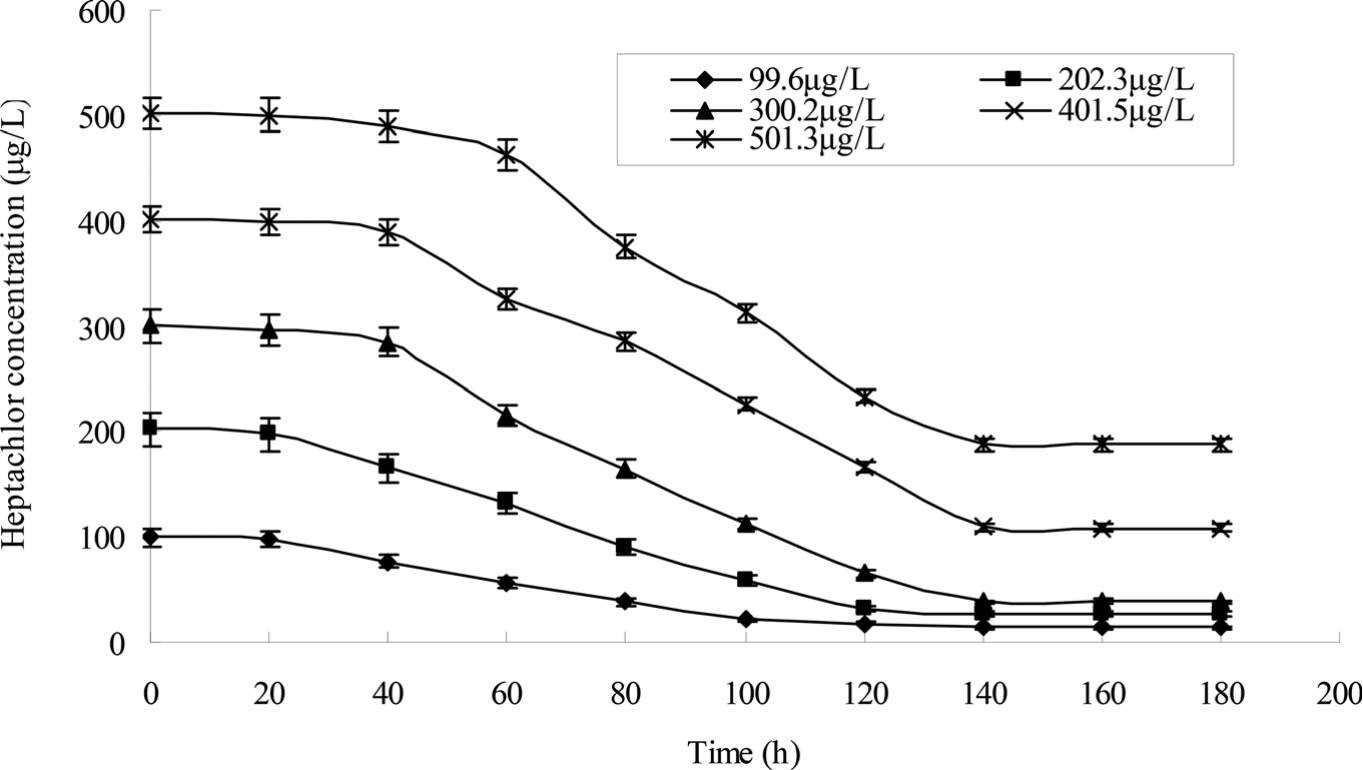
Degradation curves at different heptachlor initial concentrations.

**Fig. 13:**
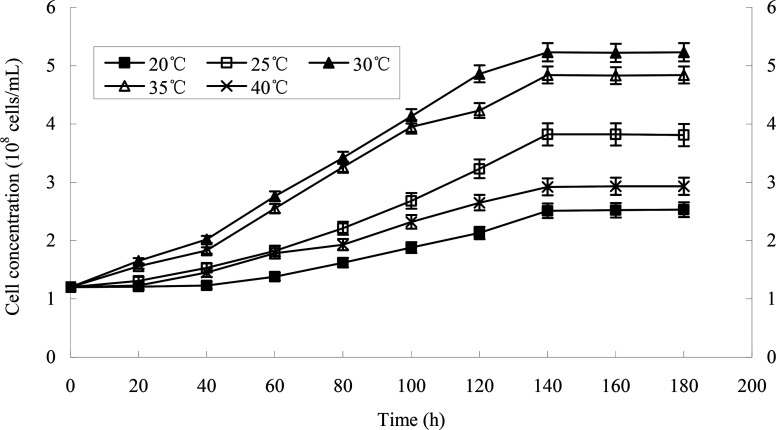
The growth curves of strain H at different temperatures.

**Fig. 14:**
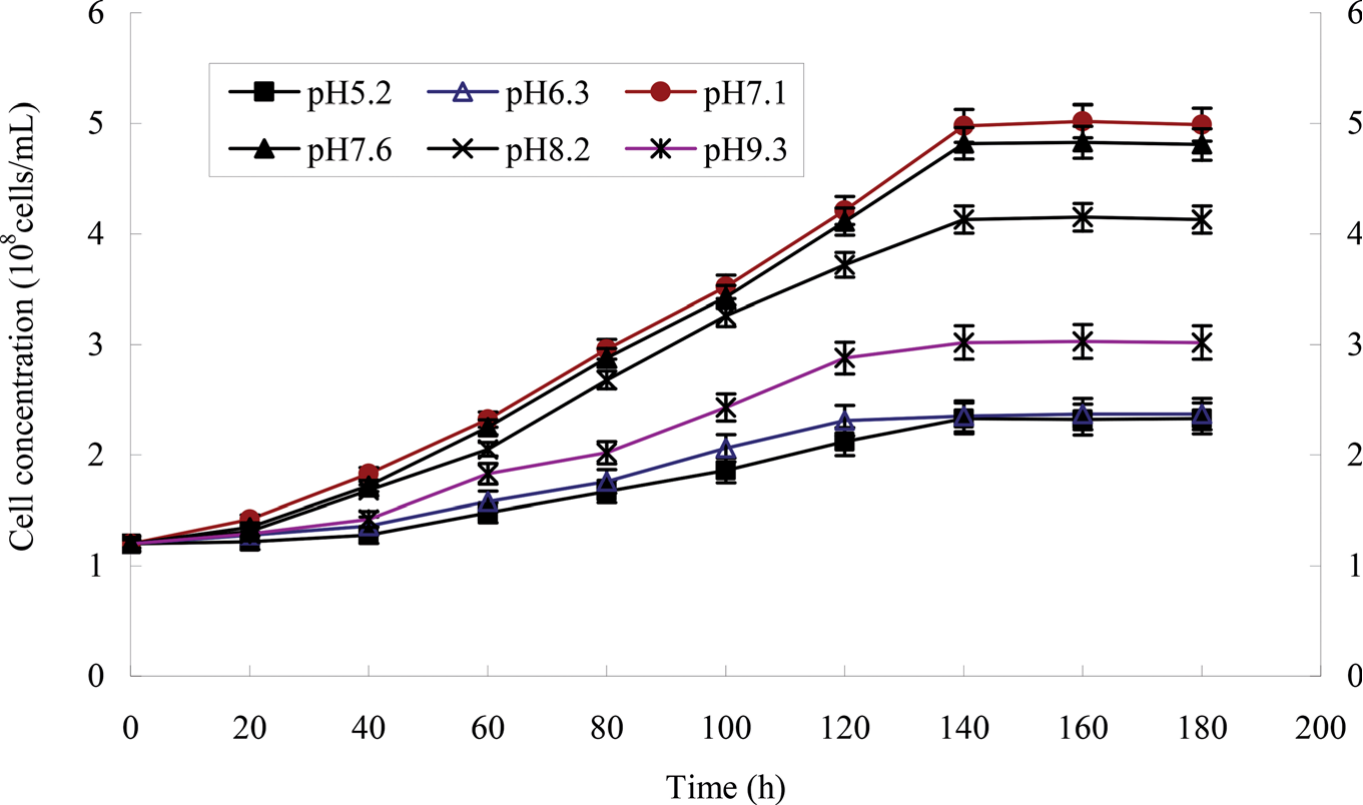
The growth curves of strain H at different pH levels.

**Fig. 15:**
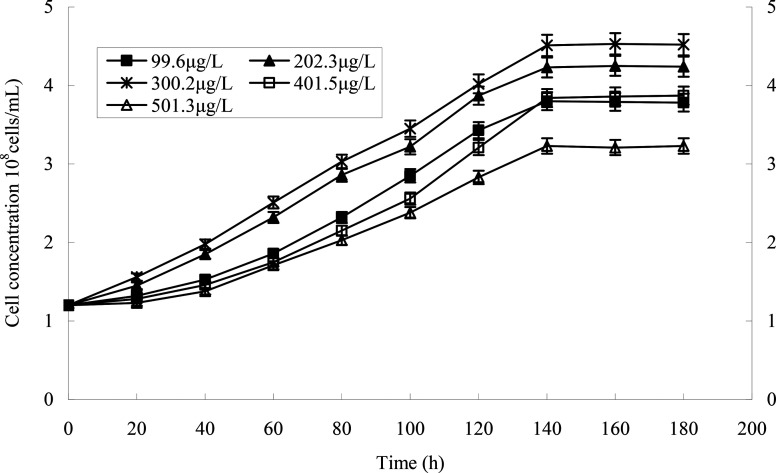
The growth curves of strain H at different heptachlor concentrations.

In previous studies, soil microorganisms transform heptachlor by epoxidation, hydrolysis, and reduction.^21^ When the compound was incubated with a mixed culture of organisms, chlordene (hexachlorocyclopentadiene, its precursor) formed, which was further metabolized to chlordene epoxide. Or heptachlor was first hydrolyzed and then dehydrated to form a heptachlor epoxide. Other metabolites include 1-hydroxychlordene, 1-hydroxy-2,3-epoxychlordene. Soil microorganisms hydrolyze heptachlor to give ketochlordene.^22^ Rats metabolize heptachlor to the epoxide 1-*exo*-1-hydroxyheptachlor epoxide and 1,2-dihydroxydihydrochlordene. When heptachlor epoxide was incubated with microsomal preparations from liver of pigs and from houseflies, the products found were diol and 1-hydroxy-2,3-epoxychlordene.^23^ Metabolic scheme in rats shows two pathways with the same metabolite. The first involves the following scheme: heptachlor ⇒ heptachlor epoxide ⇒ dehydrogenated derivative of 1-*exo*-hydroxy-2,3-*exo*-epoxychlordene ⇒ 1,2-dihydrooxydihydrochlordene. The second involves: heptachlor ⇒ 1-*exo*-hydroxychlordene ⇒ 1-*exo*-hydroxy, 2,3-*exo*-epoxychlordene ⇒ 1,2-dihydrooxydihydrochlordene.^24^

However, in this study, 1-hydroxy-2,3-epoxychlordene and 1,2-dihydroxydihydrochlordene were not detected. This may be due to the instability of the products of heptachlor epoxide hydrolysis, which could be rapidly converted into other trace metabolites, or the presence of chlorine atoms on the C2 position of heptachlor epoxide, which affected the hydrolysis of the oxygen ring.

It can also be seen from Fig. 7 that the concentration of 1-hydroxychlordene and heptachlor epoxide also gradually decreased over time. When the degradation reaction time reached 180 h, the concentration of 1-hydroxychlordene and heptachlor epoxide decreased to about 8.3 μg L^−1^ and 17.2 μg L^−1^, respectively. This may be due to the enzymatic activity of microorganisms, resulting in metabolites of heptachlor being further degraded. Enzymes are the proteins synthesized by living cells and act as efficient catalysts for their specific substrates. They are biological catalysts for most reactions in the organism. Microorganism could secrete and produce intracellular enzymes such as monooxygenase, and extracellular enzymes such as peroxidase.^25^ Under the catalysis of these enzymes, organic chlorine pollutants could be degraded effectively through multiple pathways. According to the analysis results of heptachlor metabolites, we concluded that the degradation and transformation of heptachlor depends on the microbial degradation properties of stain H.

The quantitative analysis of the above heptachlor metabolites shows that strain H also has certain degradation and conversion ability for 1-hydroxychlordene and heptachlor epoxide. This may be due to the further hydroxylation and substitution of 1-hydroxychlordene to produce multiple hydroxylation products,^26^ and the oxygen ring structure of heptachlor epoxide can be opened by hydrolysis reaction.^27^ Therefore, heptachlor and its metabolites were converted into further less-toxic small molecular metabolites through a series of reactions such as hydroxylation, epoxidation and dechlorination.

Based on the above analysis, we suggest that strain H has the potential for the remediation of heptachlor-contaminated soil and water at the temperature of 30 ± 0.5 °C.

Proposed metabolic pathways of heptachlor degradation by strain H is presented in Fig. 8. We propose the following two initial metabolic pathways of heptachlor by strain H. Pathway (1) hydroxylation at the C1 position of heptachlor to 1-hydroxychlordene followed by epoxidation and dechlorination to chlordene epoxide; and pathway (2) epoxidation at the C2, C3 positions of heptachlor to heptachlor epoxide, and then heptachlor epoxide was further converted by hydrolysis or dechlorination reaction. On the one hand, dechlorination reaction at the C1 position of heptachlor epoxide may be transformed into chlordene epoxide. On the other hand, hydrolysis reaction at the C2, C3 position of heptachlor epoxide may be degraded into heptachlor diol.^28^

## Conclusions

In this study, we isolated and screened a heptachlor-degrading microorganism named strain H, which was able to effectively degrade heptachlor at the temperature of 30 ± 0.5 °C. Experiments showed that strain H can grow using heptachlor and its degradation products as its carbon source. Strain H was identified as a novel Gram-negative, short rod-shaped, single-cell bacterial strain. When heptachlor degradation rate reached the maximum, 88.2% at 130 h, identification of the heptachlor metabolites by GC/MS showed that the main degradation products were 1-hydroxychlordene, heptachlor epoxide and chlordene epoxide. The bioconversion mechanisms of heptachlor was accomplished by hydroxylation, epoxidation, and dechlorination at the C1 position, as well as epoxidation, hydrolysis or dechlorination reaction at the C2, C3 positions. With the degradation time prolonged, heptachlor and its metabolites could be further converted into less-toxic small molecular metabolites. Thus, strain H has the potential for the remediation of heptachlor-contaminated soil and water.

## Conflicts of interest

There are no conflicts to declare.

## Supplementary Material
